# Proposed validation stages for MPs extraction from edible mussels (*Mytilus galloprovincialis*)

**DOI:** 10.1016/j.heliyon.2024.e32212

**Published:** 2024-06-06

**Authors:** G. García Rosales, F. Oberhaensli, C.M. Alonso-Hernández, L.C. Longoria-Gándara

**Affiliations:** a IAEA Environment Laboratoires, 4 Quai Antoine 1er B.P. 800, MC-98000, Monaco; b TECNM/Instituto Tecnológico de Toluca-DEPI. Av. Tecnológico s/n. Colonia Agrícola Bellavista Metepec, C. P. 52149, Mexico; c Division for Latin America/Department of Technical Cooperation International Atomic Energy Agency, Wagramer Strasse 5, P.O. Box 100, A-1400, Vienna, Austria

**Keywords:** Mussels, Microplastics (MPs), Validation, Corolase, LDIR

## Abstract

The potential presence of microplastics (MPs) in seafood products presents significant health concerns, demanding the adoption of standardized and validated methodologies. In this study, we introduce a validated method and an innovative technique for extracting MPs from mussels using an oxidizing agent, Corolase enzyme, and a surfactant, thus eliminating the need for mechanical agitation. Evaluation of the extraction process focused on three critical parameters: recovery percentage, repeatability, and chemical integrity, along with color stability. To ensure precision and reliability, low-density infrared spectroscopy (LDIR) was employed to analyze the effect of spectrum quality (Q). Ultimately, this methodology was applied to identify MPs in commercial mussels, with results showcasing the viability of the proposed validation stages for MPs extraction, maintaining MPs integrity with high recovery percentages.

## 1 Introduction

Plastics, originating in the 19th century, serve a multitude of purposes across industries and everyday life [[1], [2], [3], [4]]. Nonetheless, the escalating demand for plastics has led to a mounting accumulation of waste. Projections indicate that by 2050, approximately 15 billion metric tons of plastic waste will have been generated [5]. Over time, plastics degrade into microplastics (MPs) minute particles ranging from 1 μm to 5 mm. These MPs stem from diverse sources, including the breakdown of larger plastic items like bottles and bags (secondary MPs) and the direct release of products containing microbeads such as personal care products (primary MPs) [[6], [7], [8]]. The presence of MPs, raises significant environmental concerns due to their persistent nature, widespread distribution, and potential adverse effects on ecosystems and human health.

Recent studies have detected MPs in air, soil, water, and polar regions [[9], [10], [11], [12]]. Plastic pollution has notably impacted oceans, with 80 % of litter originating from land-based sources, posing a threat to marine species. The remaining 20 % is attributed to activities at sea [13]. Aquatic organisms, including fish, birds, and marine mammals, are vulnerable to ingesting MPs, often mistaking them for food, thereby introducing these particles into their bodies and the food chain [[14], [15], [16], [17]]. Furthermore, MPs can bioaccumulate as larger species consume smaller ones [18]. These MPs may contain various additives, such as UV stabilizers, flame retardants, and antibacterial particles, constituting up to 4 % of their composition [[19], [20], [21], [22], [23]]. They also have the ability to adsorb organic and inorganic contaminants on their surfaces, thereby increasing their toxicity [24] (Sauda et al., 2023). Currently, more than 690 marine species have been affected by MPs, with up to 10 different types identified in the digestive tract and gills of various organisms, including bivalves [[25], [26], [27], [28]].

While shellfish, such as mussels, oysters, and clams, offer numerous health benefits, including being excellent sources of high-quality protein and essential nutrients such as omega-3 fatty acids and minerals [29,30], it's crucial to be aware of possible contaminants. The World Health Organization (WHO) and the Food and Agriculture Organization of the United Nations (FAO) recommend limiting seafood consumption to one or two servings per week [31]. To address MPs' issue in food safety, regulatory agencies and the food industry have been implementing monitoring and research programs to better understand the sources, levels, and potential risks of MPs contamination in food. Although there are currently no specifically legislated maximum residue limits for MPs [32], the European Food Safety Authority [33] recommends identifying and quantifying MPs' presence in foods [34]. Furthermore, some countries have taken measures to reduce plastic use to mitigate the generation of these particles and, consequently, their presence in food [35]. Numerous studies have explored various methods to extract and identify MPs (oxidative, basic, acidic, enzymatic) [36]. However, discrepancies arise due to the lack of standardization in analytical, processing, and sampling techniques, making direct comparisons challenging.

This study introduces a validated method and a novel approach for MPs extraction, utilizing an oxidizing agent, the enzyme Corolase®, and a surfactant to efficiently digest mussels (*Mytilus galloprovincialis*), eliminating the need for mechanical agitation. Our evaluation of the extraction process focused on three crucial parameters: i) recovery percentage, ii) repeatability and chemical integrity, and iii) color stability. For accurate and reliable MPs identification, we employed low-density infrared spectroscopy (LDIR). Through our methodology, we analyzed MPs in commercial mussels.

## 2 Methodology

This methodology employs an oxidizing agent, the Corolase enzyme, and a surfactant. Notably, the extraction method outlined in this study eliminates the need for agitation. A detailed description of each experimental stage is provided.

### 2.1 Sample preparation

The sample preparation process is pivotal for ensuring precise handling and treatment to achieve reliable and accurate results. In this study, mussels sourced from a supermarket were utilized, and the detailed procedures for sample preparation are outlined below:

a) Selection of mussels: Mussels with closed shells from a 1.5 kg commercial boat were exclusively chosen for the study. This criterion ensures that the mussels are alive and in optimal condition, with those exhibiting open or damaged shells excluded to uphold sample integrity.
b) Washing: The selected mussels underwent a comprehensive washing process using pre-filtered demineralized water with a pore size of 0.22 μm. This critical step is essential to eliminate impurities and external contaminants from the mussel shells, ensuring their cleanliness. The thorough washing process is vital to prevent contamination during the mussel tissue extraction phase.
c) Freezing: Following the washing step, the mussels were rapidly frozen at −22 °C. Freezing is instrumental in preserving the integrity of the mussels and preventing potential sample degradation before analysis, ensuring sample stability during storage. During the sample preparation phase, the mussels were treated frozen to prevent part of the shell content from being lost upon thawing. By meticulously following these detailed sample preparation steps, the integrity of *Mytilus galloprovincialis* mussels is maintained, and the impact of potential contaminants or biases is minimized. This approach guarantees the production of reliable and accurate results in subsequent analyses.

### 2.2 Extraction process and identification of MPs

#### 2.2.1 Mussel dissection

From the package of commercial mussels, 25 specimens of similar size (approximately 4 ± 1 cm) and nearly uniform weight were chosen randomly. Utilizing precision tools such as a scalpel and metal tweezers, the contents of the mussel shells were meticulously extracted and amalgamated to create a composite sample from the 25 mussels. Following this, 2g pull samples were individually transferred to beakers to initiate the digestion process.

#### 2.2.2 Digestion process

To initiate the digestion process, 2 mL of 35 % Merk® H_2_O_2_ (hydrogen peroxide) was introduced into each beaker containing the mussel tissue. To maintain a contamination-free environment, the beakers were securely covered with watch glasses and aluminum foil, creating a sealed setting. This precautionary step was repeated to ensure maximum protection against contamination. Subsequently, the beakers were carefully placed in an oven and maintained at a controlled temperature of 50 °C for 1 h. Following the incubation period, 2 mL of Corolase 7089 Enzymes® was added to each beaker, facilitating further digestion. The samples were then incubated for an additional 3 h at 50 °C, allowing the enzymes to break down complex components effectively. After this stage, 7 mL of 8 % Trizma® basic solution (pH = 9) (Sigma Aldrich®) was meticulously introduced into each beaker to guarantee thorough mixing. The samples were incubated for 3 h more at 50 °C, optimizing the digestion process and ensuring efficient decomposition of organic matter.

To proceed, 40 mL of 2 % SDS (Sodium Dodecyl Sulfate, Sigma Aldrich® ≥99 %) were added, ensuring correct mixing and contact with the samples. Then, the beakers were left undisturbed for 19 h at 50 °C. After the digestion process, the solution was filtered using a stainless-steel mesh filter with a pore size of 25 μm. The vacuum pump speed is carefully adjusted to (∼10 kPa) to prevent the loss of MPs due to excessive suction force during the vacuum.

#### 2.2.3 Separation of MPs by density difference

It's crucial to emphasize the significance of separating microplastics based on density differences, especially considering that mussels often harbor sand particles and various materials depending on their environment. Since mussels are filter feeders, they intake water that may contain diverse materials alongside microplastics.

Thus, the particulate matter containing MPs retained in the stainless-steel filter from the previous step is carefully transferred to a 100 mL separating funnel, along with 50 mL of Merk® sodium iodide (NaI) solution (ReagentPlus ≥99 %), characterized by a density of 1.6 g/mL. To ensure complete MPs recovery from the stainless-steel filter (pore size of 25 μm; diameter = 25 mm), it undergoes four washes with NaI solution, with the liquid transferred using a pipette to the separating funnel. The separating funnels are securely sealed and left undisturbed for 36 h to facilitate effective MPs separation. Following this waiting period, MPs are isolated from the supernatant by gradually opening the separatory funnel valve until the NaI solution volume is minimized. It's crucial to highlight that certain MPs might adhere to the funnel walls, necessitating the washing of the walls with a 10 mL prefiltered ethanol solution (1:1 v/v) to recover them.

A notable advantage of using NaI solution is its reusability through pH and density adjustments. To ensure its optimum performance before each subsequent use, the solution must be filtered through a membrane with a pore size of 0.22 μm. This filtration process effectively retains particles in the solution, preventing interference with sample separation.

#### 2.2.4 Collecting MPs for later identification: filtration process

**Fig. 1:**
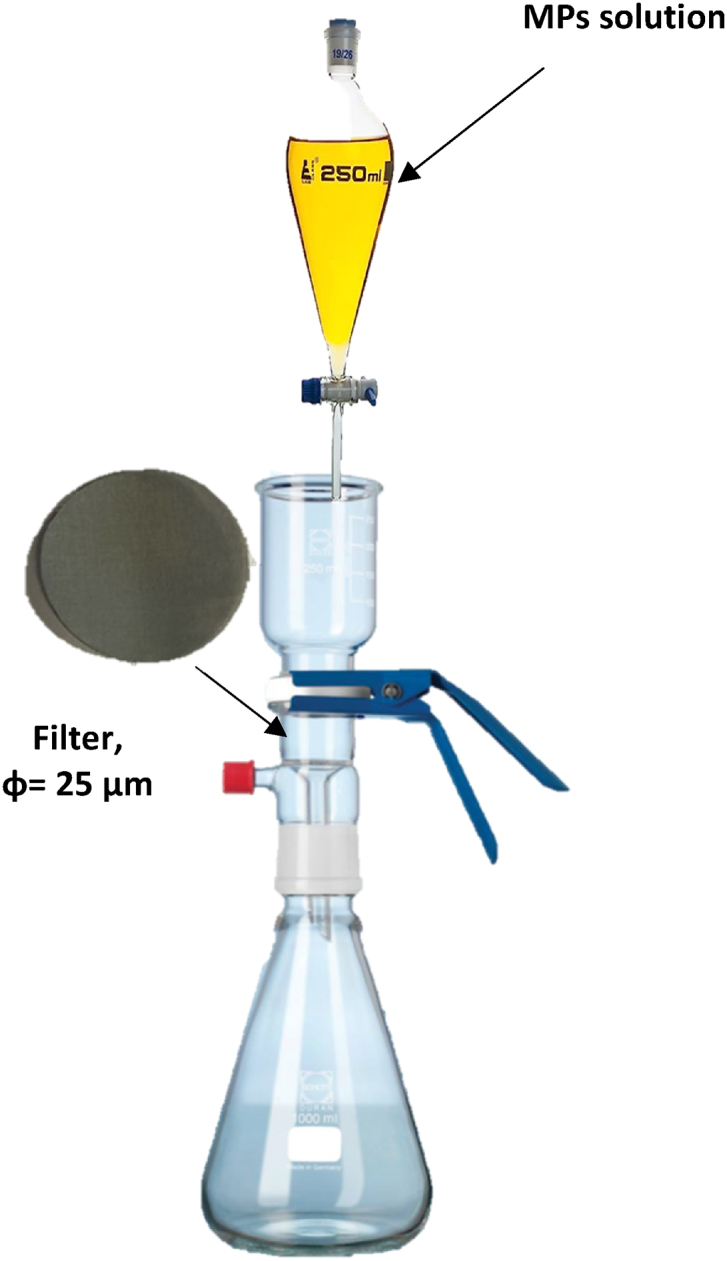
Filtration system.

In the preceding section, we delineated the method for separating microplastics based on their density disparities, leading to a residual liquid containing the microplastics in solution. This residual liquid is subsequently subjected to filtration through a system (refer to Fig. 1) equipped with a stainless-steel filter featuring a 25 μm pore size (diameter = 25 mm). Throughout this filtration procedure, a 1:1 ethanol solution is utilized to ensure both the recovery of MPs and the elimination of NaI residues.

In the following step, the material gathered on the metal filter is moved to a 50 ml beaker and subjected to ethanol solution (1:1 v/v) washing to achieve the thorough recovery of all MPs. Subsequently, a new filtrate is created utilizing gold filters (PETG, gold-coated membrane filters, pore size of 0.8 μm, and 25 mm of diameter), and the identification of MPs is conducted through LDIR.

#### 2.2.5 Efficiency of digestion

To assess the efficiency of the digestion process, the stainless-steel filters were weighed before and after the filtration procedure. By employing Equation (1) [37], the efficiency was computed based on the weights recorded prior to and following the digestion process of the filters. This protocol was replicated five times to ensure reliable and valid outcomes.

(Eq 1) $$\%\;De=\left(\frac{Wi-\left(W_{a}-W_{b}\right)}{W_{i}}\right)x100$$

where: a) De is the digestion efficiency (%), b) $W_{i}$ = Initial weight of biological material, c) $W_{a}$ = is the weight of dry filter after filtration and d) $W_{b}$ = is the weight of dry filter before filtration.

#### 2.2.6 Identification of MPs

##### 2.2.6.1 LDIR

The LDIR method is a valuable technique for identifying and quantifying MPs in the size range of 20–100 μm. It relies on infrared spectroscopy, which involves the interaction between infrared radiation and the molecular vibrations of MPs particles. In this study, the Agilent® 8700LDIR Chemical Imaging system was utilized to analyzer the samples. To ensure consistent and reliable measurements, the samples were carefully positioned on a holder under the infrared beam, specifications for the embedded lasser are: maximum power: 9.6 mW, pulse rate:500 kHz, pulse duration: 80ns, wavelength: 5–11 μm. Maximum beam divergence: cono half angle >45° coming to a focus in the center of the sample compartment and diverging thereafter. The instrument was calibrated using a standard reference material and the wavelength range 975–1800 cm^−1^ and the resolution were adjusted according to the specific analysis requirements. During data acquisition, a dedicated method was employed to achieve the desired signal-to-noise ratio and ensure high-quality data. Following data acquisition, the collected spectral data underwent processing and analysis using (Clarity 1.5) specialized software. The team makes a comparison between the library and the spectrum of the sample, only the results with a coincidence greater than 75–90 % were considered.

### 2.3 Validation process

#### 2.3.1 Recovering

Validating the recovery process is crucial for ensuring the effective retrieval and measurement of MPs. Control experiments are essential, employing known quantities and types of MPs particles to evaluate the method's effectiveness in recovering introduced MPs. In this study, five replicates were prepared for each size range of fluorescent spherical polyethylene particles (Cospheric® Innovations in microtechnology) with diameters of 32–38 μm, 53–63 μm, and 106–125 μm, respectively. Polyethylene was chosen for its widespread presence as a common environmental contaminant. Each set of five samples for each particle size consisted of 2 g of mussel tissue spiked with 25 fluorescent beads to establish a predetermined quantity of MPs for evaluating the recovery process. Subsequently, the spiked samples underwent the proposed extraction methodology, involving digestion, filtration (using stainless steel filters with pore sizes of 25 and 100 μm, depending on the particle size to be separated), and density-based separation. These procedures are crucial for isolating and recovering MPs from mussel samples. After the recovery process, the collected MPs particles were quantified using a Leica® stereoscope and a Nightsea® B-Royal Blue UV lamp (λe = 440–460 nm).

The efficiency of MPs recovery was computed by taking the amount of MPs recovered after the extraction process $\left({MPs}_{f}\right)$, multiplying it by 100 to express it as a percentage, and then dividing this result by the known initial quantity of MPs $\left({MPs}_{i}\right)$. The recovery data were analyzed to assess the effectiveness of the method employed in the study (Eq. (2)).

(Eq 2) $$\%\;R=\left(\frac{\left({MPs}_{f}\;X\;100\right)}{{MPs}_{i}}\right)$$

#### 2.3.2 Assessing MPs integrity: Stability of coloration and chemical effects

Assessing the chemical integrity and color of MPs is important to understand their composition and potential effects on the environment. Therefore, in order to evaluate the effect of the extraction process on the color of MPs and their chemical stability, in this study spheres of different colors were selected and divided into control groups and test groups. The test groups underwent the extraction process, while the control groups were untreated and served as reference. The colored spheres were subjected to the extraction process using the method described in section 2. After the extraction process, a color analysis of the MPs samples was carried out using the color space chromaticity diagram, where the values were obtained, such as brightness (L*) and color components (a* and b*).

On the other hand, to evaluate the chemical stability of MPs, samples were prepared using 2 g of mussel tissue to which reference plastic particles were added: low-density polyethylene (LDPE), polypropylene (PP), and polyamide nylon from Goodfellow® Cambridge, England. The plastic particles, initially sized at 2–3 mm, were reduced to particles ranging from 30 to 150 μm, chosen for their prevalence in nature. Subsequently, the digested plastic samples were analyzed using LDIR spectroscopy to identify potential changes in the chemical composition of the MPs following the extraction process. Once the methodology was validated and optimized, it was applied to commercial mussels from the Atlantic Ocean with the intention of determining the concentration of MPs per gram of mussel.

### 2.4 Quality controls

Due to the importance of strictly applying QA/QC quality controls and in order to minimize contamination, different control measures were taken. To begin with the experimentation, all the materials were washed, and heat treated for 2h at 450 °C. To eliminate the presence of fibers or plastic fragments, utensils made of this material were not used during the experimentation. Blanks were prepared as control media to be considered in the results and sampling was carried out in the study area to identify the degree of contamination by MPs or fibers in the laboratory where the samples were processed. For the preparation of the solutions, milliQ water was used, which after its preparation was again filtered to avoid the presence of fibers or impurities foreign to the samples that could be in the environment. To carry out the experiments, a cotton gown and clothing were used, and each sample was carefully covered with a watch glass and aluminum foil during processing, air currents were avoided to avoid contamination of the samples. The stainless-steel filters were treated with acetone and ultrasound. It is worth noting that the filters were inspected with a stereoscope to ensure their cleanliness.

## 3 Results and discussion

### 3.1 Digestion process efficiency

Establishing extraction conditions without causing damage to MPs is a challenging task due to the intricate composition of mussels. Mussels, as marine organisms dwelling in aquatic environments, possess a high-water content [38]. Moreover, they comprise fibrous collagen structures with proteins containing up to 19 different types of amino acids, along with fats, calcium carbonate, and minerals serving as chelating agents. These elements reinforce the fibers and contribute to their rigidity [39]. Additionally, the intestines of mussels contain sediments and plankton with complex structures, making them resistant to destruction during MPs extraction [40,41]. Therefore, to establish the methodology for MPs extraction in this study, various factors such as the enzyme amount, agitation effect, reagent quantities and concentrations, and pH were evaluated. An essential part of the extraction process involves freezing the mussel tissue to alter the lipid and protein structure, making the tissue more susceptible to tearing. The next step is to add H_2_O_2_, which aims to fractionate the tissue and degrade organic matter [42], assisting in breaking down the protein structure. This step is carried out for 1h to avoid damaging the polymeric structure of the MPs [43]. Next, the enzyme Corolase is introduced into the system, which, through hydrolysis, breaks the peptide bonds of the proteins, forming carboxylic acid and water, leading to acidification of the reaction medium. To maintain a constant pH and enhance the efficiency of Corolase, a Tris buffer solution is used to control the pH near to 8 in the system [44]. Later, SDS is added to break down shorter protein chains and dissolve the remaining fats, primarily triglycerides found in the digestive gland, mantle, and gills [45]. It's important to note that the use of Corolase not only aids in the digestion process but also plays a crucial role in preserving the integrity of the plastics during extraction. However, the effectiveness of tissue dissolution may vary depending on factors such as mussel species, age, and the digestion methods used to effectively break down the biological tissues and minimize the impact on the MPs. In this study, using the proposed oxidative-enzymatic method, a digestion efficiency of 99.7 ± 0.8 % was achieved. Some small tissue particles were observed on the filters; however, these did not interfere to quantify MPs by LDIR spectroscopy. This result compares favorably with the efficiencies reported by Friesen et al. [46], who achieved 97.7 ± 0.2 % using pancreatic enzyme and Tris; Löder et al., [42]. who reached 98.3 ± 0.1 % employing three different enzymes and SDS in the process; and Rist et al. [47], who obtained a digestion efficiency of 99.44 % using Proteinase K and Catarino et al. [48], who used Corolase. It's important to emphasize that the digestion process does not entail shaking the sample. However, if deemed necessary, the digestion vessel can be gently manually agitated a few times, though such action is generally not required.

### 3.2 Validation

#### 3.2.1 Recovering

**Fig. 2:**
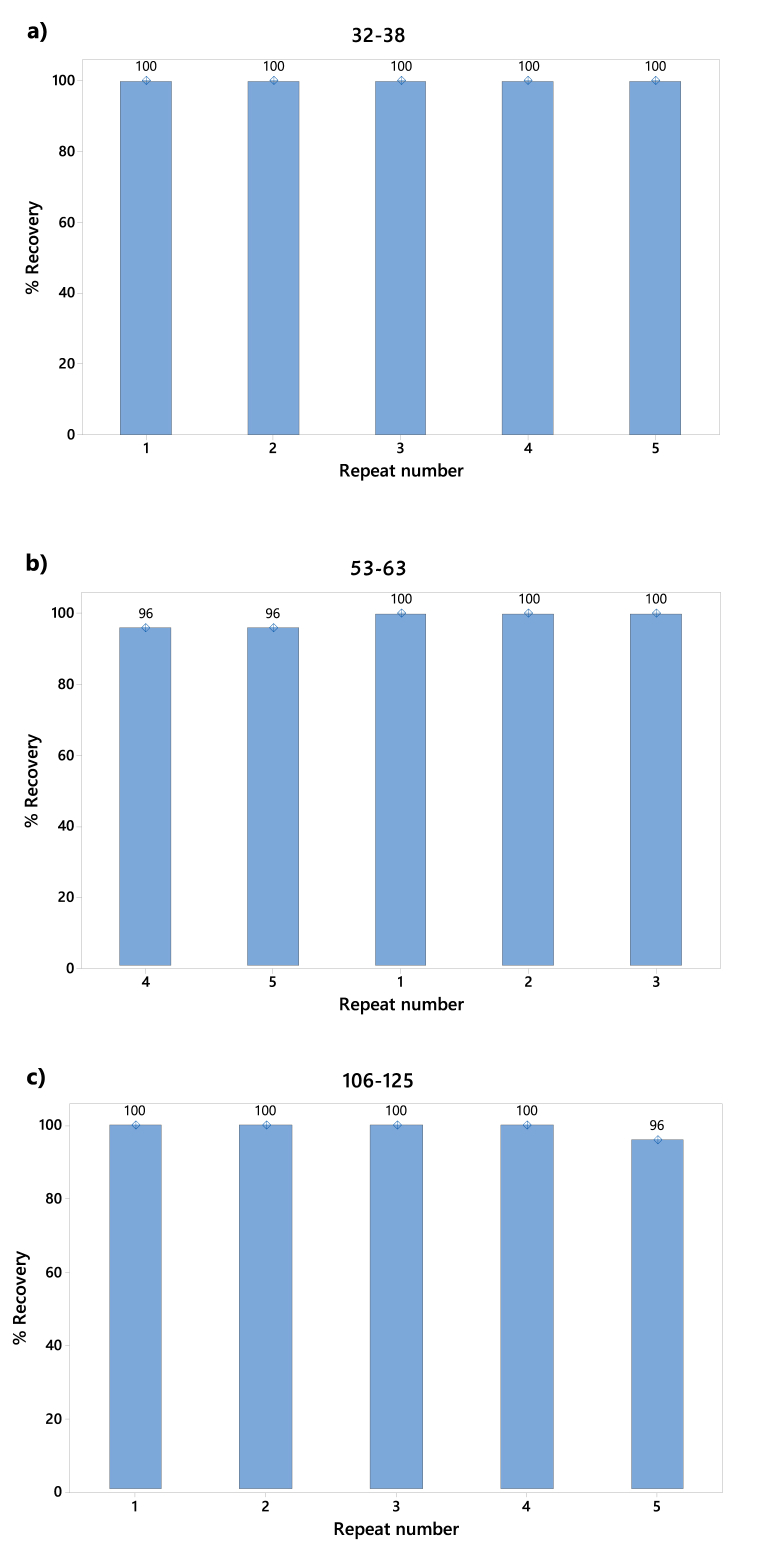
Recovery of fluorescent spherical polyethylene MPs applying the extraction process: a) 32–38 μm; b) 53–63 μm and c) 106–125 μm.

Fig. 2 a-c) presents the outcomes, revealing that the most substantial recovery rates were attained with the smallest particles (100 %), demonstrating negligible divergence when contrasted with larger diameter counterparts. It is imperative to underscore that, drawing from our experiential insight, excessive vacuum force surpassing 10 mPa during recuperation via the filter could potentially lead to the passage of minute particles through the filter mesh due to the inherent plastic material flexibility. Across all scenarios, recovery percentages spanned from 96 % to 100 %. These values transcend those documented in alternative investigations utilizing NaCl as the segregating medium [49]. This behavior implies that the extraction procedure maintains the integrity of the PET surface, producing a more efficient separation in the NaI.

Nonetheless, vigilance during the recovery phase is of paramount importance, as MPs possess the propensity to adhere to sundry surfaces, encompassing separatory funnel walls, components of the filtration setup, and metallic sieves, thereby potentially culminating in an underestimation. Antecedent research has also underscored this intricate aspect during the transition of particles from the supernatant to the filtration setup [49,50]. Hence, a comprehensive cleansing of these surfaces is strongly advised to ensure the exhaustive retrieval of MPs.

#### 3.2.2 Impact of digestion process on color and chemical structure of MPs

**Fig. 3:**
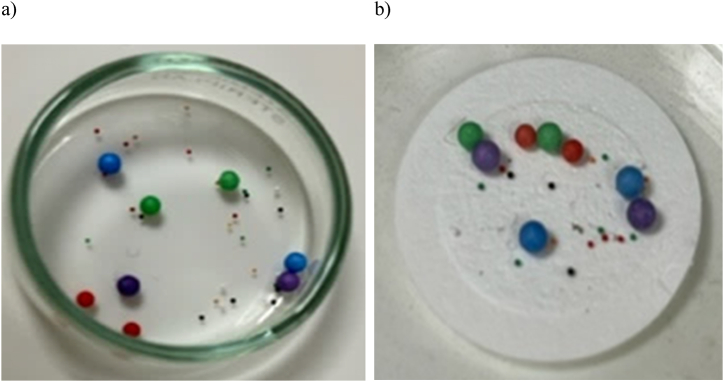
Effect of the digestion process on the color stability of polystyrene spheres: a) before and b) after. (For interpretation of the references to color in this figure legend, the reader is referred to the Web version of this article.)

In Fig. 3 a-b), the spheres subjected to the digestion process are shown, where it can be observed that their color remained unchanged. This is demonstrated by the analysis of chromatic coordinates and luminosity values: Deep blue (a* = +70, b* = −70, L* = 17), vibrant red (a* = ^+^87, b* = ^+^87, L* = 70), lime green (a* = ^-^60, b* = ^+^60, L* = 58), radiant violet (a* = ^+^58, b* = ^-^58, L* = 32), rust yellow (a* = ^-^63, b* = ^+^63, L* = 74), black and white; all retained their original tones, indicating that the digestion process does not impact the color of the MPs. This suggests that the extraction process does not promote degradation or chemical modification through depolymerization, as has been observed in some non-enzymatic studies [37].

Our findings align with the results reported by Karlsson et al. [51], who proposed an extraction method using Protease where the color and properties of the MPs were similarly unaffected. Despite the use of H_2_O_2_ in our study, known for its potential to damage MPs color [37,49], no negative effects were observed. It is hypothesized that the Corolase enzyme could form a protective layer around the MPs, shielding them from the environment.

### 3.3 MPs detection and quantification in mussels

**Fig. 4:**
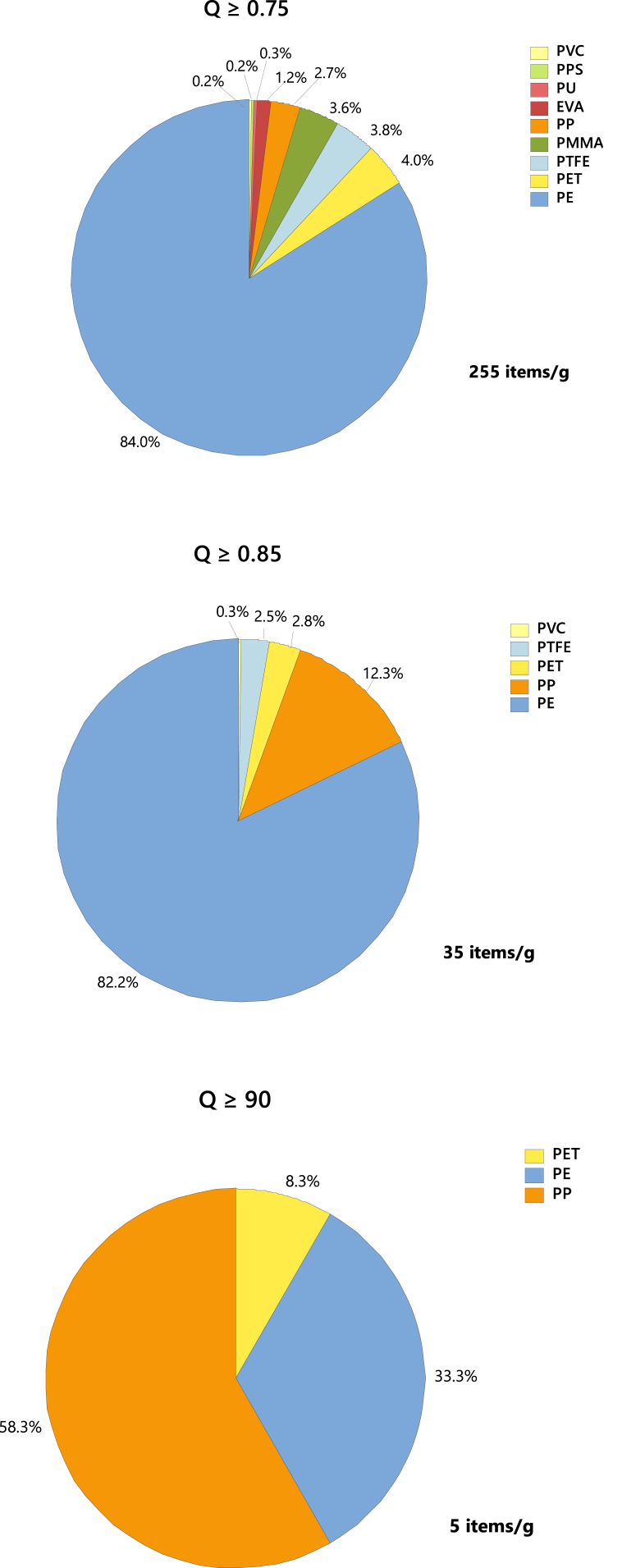
Identification of MPs in mussel samples at different Q values: a) Q = 0.75; b) Q = 0.85 and c) Q = 0.90.

Filters containing MPs, along with control and reference blanks, were analyzed using LDIR. The control blanks exhibited a limited number of fibers within the working area, while the reference blank, simulating mussel tissue digestion, was used to adjust the abundance of each particle type in the samples, considering only those identified as MPs. Nine different polymer types were identified (Q = 0.75) by LDIR: polyethylene (PE), ethylene-vinyl acetate (EVA), polyethylene terephthalate (PET), polymethyl methacrylate (PMMA), polypropylene (PP), polytetrafluoroethylene (PTFE), polyurethane (PU), polyvinyl chloride (PVC), and phenyl polysulfide (PPS) (Fig. 4). The prevalence of MPs in the samples revealed significant variability, depending on the criteria used to assess spectrum quality and database comparisons. As the strictness of the criteria increased, the number of particles considered in the study markedly decreased. This trend is clearly illustrated in Fig. 4 a-c): with a quality threshold of ≥0.9, the particle count per gram of mussel tissue stood at 5; this count rose to 35 particles/g with a threshold of ≥0.85 and surged further to 255 particles/g with a threshold of ≥0.75. The absence of a standardized criterion has resulted in substantial inconsistencies in reported findings across existing literature. While Hansen et al. [52] advocate for a Q value of 0.9 to mitigate false positives, other studies have adopted lower thresholds [53,54]. To address this variability, the present study employs three confidentiality levels: low (Q = 0.75), medium (Q = 0.85), and high (Q = 0.90), as specified by the equipment provider (Agilent®). Fig. 4) outlines the prevalence of MPs under different quality thresholds (Q). With Q ≥ 0.75, the prevalence hierarchy is as follows: PE (84 %) > PET (4 %) > PTFE (3.8 %) > PMMA (3.6 %) > PP (2.70 %) > EVA (1.2) > PU (0.3 %) > PVC (0.2 %) ≈ PPS (0.21 %). Notably, PE, PET, PETFE, PMMA, and PP exhibit the highest prevalence percentages and have been associated with identifiable additives [55,56]. When Q ≥ 0.85, the distribution shifts to PE (82.24 %) > PP (12.3 %) > PET (2.8 %) > PTFE (2.5 %) > PVC (0.30 %), with EVA, PMMA, PU, and PPS becoming less prominent. Within this context, the most abundant MPs are PE, PP, and PET. With Q ≥ 90 %, PP (58.3 %) emerges as the predominant polymer, followed by PE (33.3 %) and PET (8.3 %). It's evident that within medium and high confidentiality parameters, PE, PP, and PET constitute the primary constituents in terms of abundance (22 ± 0.68 MPs mussel^−1^; 5 ± 0.27 items g^−1^). These same polymers have consistently been detected in marine environments and Atlantic mussel specimens) [[57], [58], [59], [60]]. The prevalence of these MPs is closely linked to their exposure to marine organisms, particularly in the water column. Density differences play a crucial role: MPs denser than saltwater tend to sediment, while those with lower densities remain buoyant. However, aging and depolymerization can affect this behavior, potentially increasing MPs accessibility to marine organisms [59,61,62].

As a consequence of aging and surface alterations, discrepancies may arise between database spectra and those obtained from MPs sourced from marine organisms when subjected to LDIR spectroscopy, influenced by exposure conditions and duration. Consequently, these variations may lead to diminished quality (Q) values. Therefore, judicious selection of spectra with a moderately specific criterion is recommended for accurate quantification. While a correlation index ≥0.9 is suitable for pristine MPs analysis, a more adaptable threshold could be considered for real-world MPs. However, caution should be exercised to prevent erroneous assignments in cases of excessively low values [63].

### 3.4 Size and characteristics of MPs

**Fig. 5:**
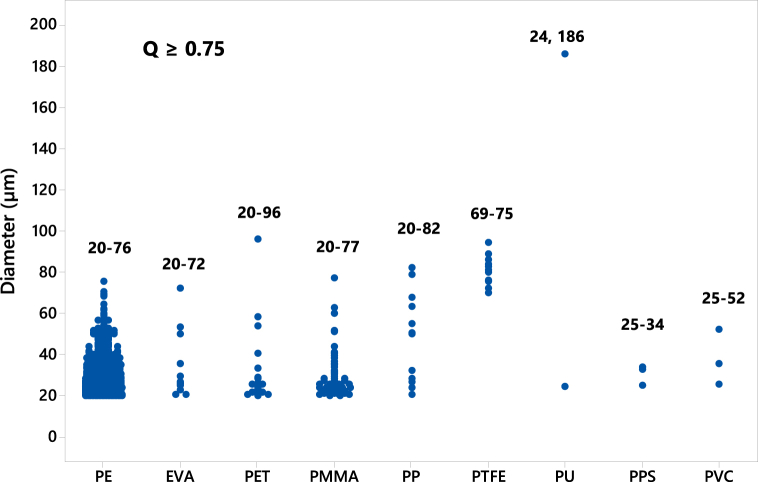
Illustrates the relationship between the diameter and abundance of MPs with a quality rating of ≥0.75.

Based on the solidity and circularity results provided by LDIR (Fig. 5), particles can be classified into pellets, non-pellets, and films or fibers. Considering the criteria previously published by Jia et al. [64], it is established that if the circularity is ≥ 0.6, the MPs particle can be considered a pellet, while values below that indicate non-pellet particles. Additionally, when solidity values are <0.3, MPs can be regarded as fibers, and when ≥0.3, they are classified as films. In this study, it was observed that the number of fibers found is limited, while approximately 69 % of the particles are identified as non-pellet films, and 31 % are categorized as pellet films.

**Fig. 6:**
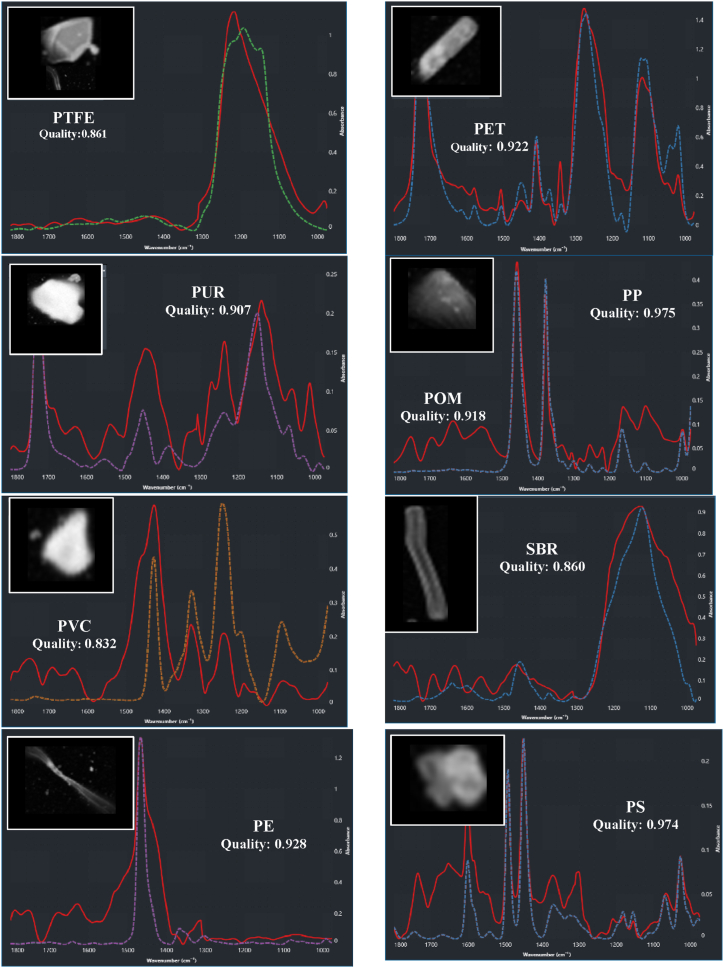
MPs identified by LDIR in mussels.

The study has identified MPs in the form of small fragments and fibers, as depicted in Fig. 6. This figure presents multiple examples illustrating the observed morphologies of the identified MPs particles (MPs).

The size of the extracted MPs falls within the range of 20–100 μm (Fig. 5), with the most predominant particle size being <60 μm. Among these, PU (24, 182 μm) exhibits the widest diameter range, followed by PET (20–96 μm), PP (20–82 μm), PE (20–79 μm), EVA (20–72 μm), PTFE (69–75 μm), PVC (25–52 μm), and PPS (25–34 μm), according to the obtained results. These data highlight that the majority of particles are very small, with only a few exceeding values of 100 μm. Consistent with Eo et al. [65], the most abundant MPs in the marine environment typically fall within the range of 1–5 mm, aligning with the findings of this study. However, variations may occur based on the mussel species under investigation [66], this difference can be seen in Table 1.

**Table 1:** MPs extraction methods and identified types in mussels, including study results.

No	Extraction and identification methods	Sample Species and Origins	Abundance (ítems/g)	Items/mussel	MPs identified	Particle size range (μm)	Reference
1	KOH 10 %, 60 °C (24 h), (μ-FTIR)	*Mytilus galloprovincialis* Qingdao, China	1.6-2.6	0.8-2.1	PVC	7–5000	[67]
2	10 % KOH 60 °C,140 rpm (24h) (μ-FT-IR)	Mytilus spp. Norway	0.97 ± 2.61	1.5 ± 2.3	PET, EVA, PP, PCV, PE	70–38700	[68]
3	Corolase® 7089 60 °C (1 night), (FT-IR)	*M. modiolus,* *Mytilus* spp UK	0.086 ± 0,031 3.5 ± 1.29	3.0 ± 0.9 3.2 ± 0.52	PET	0.2–2000	[69]
4	H_2_O_2_ 65 °C (24 h) FT-IR	*Mytilus galloprovincialis* *Viscaya, Spain*	0.26	1.62	PET, PS, PE	≤10	[70]
5	40 °C, KOH-SDS-protease-lipases, celluloses–H_2_O_2_–Fe(II)-Chitinase, ATR-FTIR and μFTIR	*Mytilus galloprovincialis* North-West Mediterranean	8.17 ± 10	18.6 ± 23.0	PE, PP, PVC, PU, PS	0.02–1000	[71]
6	Prot/Twin/Glycine/KOH 20 rpm, pH = 9, 50 °C	*Mytilus galloprovincialis* Northern Adriatic Sea	1.06-1.33 fragments 0.62-0.63 fibers		PE, PP, PET, PS, PVC	20–40	[72]
7	KOH and NaOH, or HNO_3_, or HNO_3_–H_2_O_2_, HCl orHClO_4_ 80 °C.	*Mytilus edulis* French Atlantic coast	0.23 ± 0.09	–	PP, PE	30–200	[73]
8	H_2_O_2_, 65 °C	*Mytilus galloprovincialis* Montenegrin Adriatic coast	–	–	PP, PE, PU, PS, PTFE.	–	[74]
9	KOH (10 %) over 48–72 h at 60 °C	*Mytilus galloprovincialis* Marseille Bay	4 ± 2	–	PP, PE, PS, PVC, PET	100–200	[75]
10	H_2_O_2_, 55–65 °C	*Mytilus galloprovincialis* Greek waters (Mediterranean Sea)	–	1.9 ± 0.2	PE, PP, PTFE	100–500	[76]
11	H_2_O_2_-Corolase-SDS (50–60 °C)	*M. galloprovincialis* Northern Atlantic Sea	5 ± 0.27	22 ± 0.68	PE, PP, PET, PTFE, PVC, PMMA	20–186	This work

### 3.5 Rough estimation of MPs mass

For the calculation of MPs mass per gram of mussel, the diameter of each particle and its density were taken into consideration. Initially, the volume of each particle was estimated, assuming mostly spherical geometry for the particles, with the idea that there could be compensation between particles due to their diverse geometries. However, it's important to note that this is a general approximation, although it's recognized that this approach has been previously considered in other studies [77]. Unlike these studies, our methodology incorporated both the diameter and density of each particle. For a more rigorous precision, a more exhaustive mathematical approach would be required, taking into account the individualized geometry and density of each particle, though this refinement is not the central purpose of our study.

Following our methodology, the calculation of particle volume was multiplied by their corresponding density to obtain the mass of each particle. By summing up the individual masses, the total mass was calculated. Based on the results obtained in this study, when Q ≥ 0.75, the total mass is 7 μg of MPs per gram of mussel. Similarly, for Q ≥ 0.85, the calculated mass is 2 μg, and for Q ≥ 0.90, the estimated mass is 1 μg per g mussel. The MPs abundance recorded in this study significantly exceeds the quantities previously reported by various authors using different extraction methods (see Table 1). According to EFSA, a portion of 225 g of mussels could contain up to 7 μg of MPs [77,78]. However, according to our own results, a portion of 250 mg of mussels with Q ≥ 0.75 could contain up to 65025 MPs particles with a total mass of 1890 μg. Considering Q ≥ 0.85, this number would decrease to 870 particles, with a mass of 552 μg, and for Q ≥ 0.90, 750 particles with a total mass of 250 μg of MPs. These estimates differ considerably from those reported in other studies (see Table 1). These results not only highlight the remarkable ability of mussels to efficiently filter the aquatic environment they inhabit [66,79,80] but also point out a potential issue in terms of food safety and a risk to human health. This is because MPs can accumulate contaminants on their surfaces [[22], [23], [24]]. The observed particle count in our study could be linked to the proximity of sampling sources in the Atlantic Ocean, a relationship that could be attributed to the substantial presence of MPs derived from the Atlantic garbage patch [81].

## 4 Conclusion

The potential presence of MPs in seafood raises significant concerns for human health, necessitating the adoption of standardized and validated methodologies. In this study, we introduce a validated method and an innovative approach for extracting MPs from mussels using an oxidizing agent, an enzyme, and a surfactant. The evaluation of the extraction process focused on three critical parameters: percentage recovery, repeatability, and chemical integrity, alongside color stability. To ensure precision and reliability, low-density infrared spectroscopy (LDIR) was employed to analyze the quality (Q) of the spectra. The results obtained demonstrate the viability of the methodology, as it effectively dissolves mussel tissue, facilitating MPs separation. Moreover, there is no alteration of coloration or chemical change in the MPs during the process, and the recovery percentages are very close to 100 %. This study underscores the importance of establishing spectrum quality criteria above 0.90 to ensure result reliability.

## Ethical approval

“Not applicable”.

## Consent to participate

García Rosales, C.M. Alonso-Hernández and F. Oberhaensli have collectively agreed to be listed as authors for this manuscript, which has been submitted to the Heliyon Journal. Upon acceptance, this work will advance to the publication stage.

## Consent to publish

All authors concur that upon acceptance by the Heliyon Journal, this work will proceed to publication.

## Funding

“The authors declare that no funds, grants, or other support were received during the preparation of this manuscript”.

## Data availability statement

“Not applicable”. The data supporting the findings of this study are included in this publication.

## CRediT authorship contribution statement

**G. García Rosales:** Writing – review & editing, Writing – original draft, Methodology. **C.M. Alonso-Hernández:** Conceptualization. **F. Oberhaensli:** Conceptualization. **L.C. Longoria-Gándara:** Conceptualization.

## Declaration of competing interest

The authors declare the following financial interests/personal relationships which may be considered as potential competing interests:G. Garcia-Rosales reports financial support, article publishing charges, and equipment, drugs, or supplies were provided by 10.13039/501100004493International Atomic Energy Agency, Environment Laboratoires, Monaco. If there are other authors, they declare that they have no known competing financial interests or personal relationships that could have appeared to influence the work reported in this paper.
